# Laparoscopic management of abdominal cocoon

**DOI:** 10.4103/0972-9941.40992

**Published:** 2008

**Authors:** Ramesh Makam, Tulip Chamany, Saraswathi Ramesh, Vamsi Krishna Potluri, Prasanth J Varadaraju, Pradeep Kasabe

**Affiliations:** Department of Minimal Access Surgery, AV Hospital, Bangalore, Karnataka, India

**Keywords:** Abdominal cocoon, intestinal obstruction, laparoscopy

## Abstract

“Peritonitis fibrosa incapsulata”, first described in 1907, is a condition characterized by encasement of the bowel with a thick fibrous membrane. This condition was renamed as “abdominal cocoon” in 1978. It presents as small bowel obstruction clinically. 35 cases of abdominal cocoon have been reported in the literature over the last three decades. Abdominal cocoon is more common in adolescent girls from tropical countries. Various etiologies have been described, including tubercular. It is treated surgically by releasing the entrapped bowel. We report a laparoscopic experience of tubercular abdominal cocoon and review the literature.

## INTRODUCTION

Abdominal cocoon is a rare cause of small bowel obstruction. Diagnosis of this rare condition is usually made perioperatively and the treatment of choice is surgical release of the entrapped bowel. Nine cases of tubercular abdominal cocoon have been reported in literature. We report a unique case of laparoscopic management of abdominal cocoon with tuberculous ascites.

## CASE REPORT

A 21-year-old Nepali woman was admitted with complaints of abdominal pain and vomiting of one day duration. She had colicky pain in the periumbilical region and left iliac fossa with intermittent vomiting and abdominal distension.

**Figure 1:**
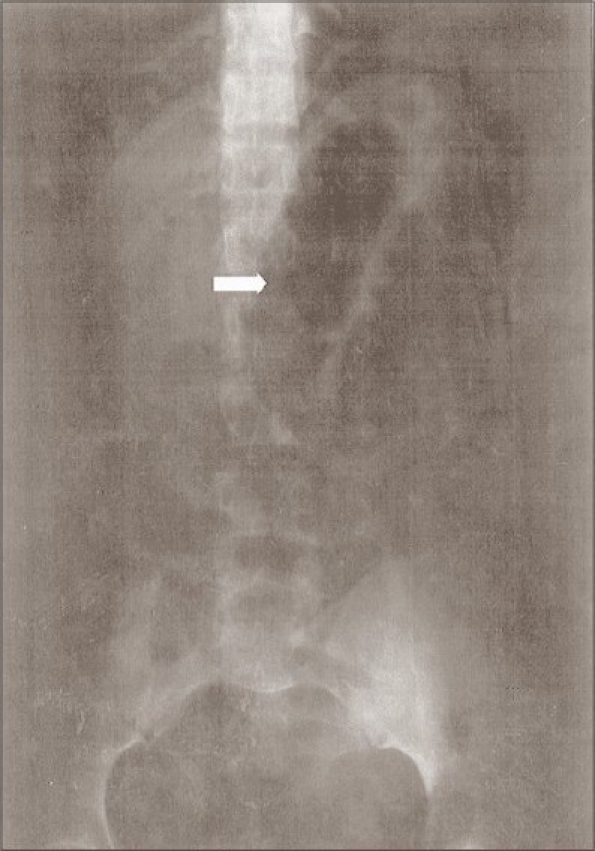
Plain X-ray abdomen showing dilated small bowel loops

On examination, the abdomen was mildly distended and tenderness was present in the left iliac fossa. A soft to firm, mobile, mass measuring 10 × 10 cm was palpable in the left iliac fossa. It was an intraabdominal, intraperitoneal mass. Bowel sounds were exaggerated. Per rectal examination was normal. Laboratory workup revealed normal findings except raised ESR of 24 mm/h. Plain X-ray abdomen revealed localized dilated small bowel loops [Figure 1]. Ultrasound abdomen revealed distended small bowel loops but no mass was detected. A clinical diagnosis of acute small intestinal obstruction was made.

**Figure 2:**
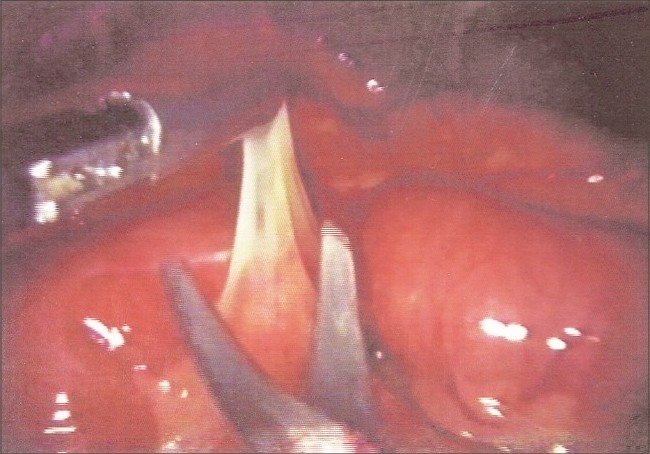
Intraoperative photograph showing excision of the thick fibrous membrane of the cocoon

Diagnostic laparoscopy was done using Hasson's technique. Accessory ports were placed in the suprapubic and left lumbar regions under vision. There was approximately 100 ml of straw colored fluid in the peritoneal cavity, which was sent for PCR-DNA for tuberculosis. The proximal small bowel was dilated and was covered by a dense, thick, whitish membrane which appeared like a cocoon. The membrane was lifted up working between it and the bowel with a scissors. Blunt tipped scissors was used with the concavity facing upwards [Figure 2]. The bowel and membrane are held separately with the graspers and the membrane is cut carefully after lifting it off from the bowel. Peritoneal biopsy was taken.

Histology of the membrane revealed fibrocollagenous tissue with congested blood vessels. Peritoneal biopsy revealed fibrocollagenous and fibro fatty tissue with mild lymphocytic infiltrate. Congested blood vessels were seen. There was no evidence of granuloma/malignancy.

Peritoneal fluid for PCR-DNA was positive for Mycobacterium tuberculosis.

## DISCUSSION

Abdominal cocoon is a rare cause of small bowel obstruction which is characterized by partial or total encasement of the small bowel by a thick fibrous membrane. Foo *et al.* described this condition in adolescent girls and named it “abdominal cocoon”. 35 cases of abdominal cocoon have been reported in the English literature.[1] We could come across nine cases of abdominal cocoon with a possible tubercular etiology in the literature.[2–4] Six cases of laparoscopic diagnosis of abdominal cocoon[5] have been reported.

Patients are usually adolescent girls who present with clinical features of intestinal obstruction. Abdominal mass may be palpable. Radiological findings are nonspecific. Barium small bowel series is suggestive when the characteristic “cauliflower sign” is detected. Definitive diagnosis of abdominal cocoon is made perioperatively.

Histology of the membranous tissue in a primary cocoon shows proliferation of fibro connective tissue with non-specific chronic inflammatory reaction. In a tubercular abdominal cocoon, histology shows caseating epitheloid cell granulomas.

In our case, the patient had abdominal cocoon encasing the proximal small bowel with fluid in the peritoneal cavity. The histology of the encasing fibrous membrane was suggestive of inflammatory pathology. There was no histological evidence of tuberculosis. Biopsy of the peritoneum was also suggestive of nonspecific chronic inflammatory response. Ascitic fluid for PCR-DNA revealed tuberculosis. Abdominal cocoon in our case - idiopathic or secondary to tuberculosis remains unresolved. Laparoscopy was diagnostic and therapeutic in this case, with less morbidity to the patient (patient was discharged on the first postoperative day). Patient was asymptomatic postoperatively as the membrane was excised. Antitubercular therapy was started.

Preoperative diagnosis requires a high index of clinical suspicion. Adolescent girls presenting with small bowel obstruction and a palpable abdominal mass without any obvious cause, the possibility of an abdominal cocoon should be considered. Excision of the membrane is the treatment of choice and the prognosis is excellent. Diagnostic laparoscopy, in this era, has a major role in the management of rarer causes of intestinal obstruction, such as abdominal cocoon. Laparoscopic treatment of abdominal cocoon needs surgical expertise, considering the risk of intestinal injury.
